# Pharmacokinetics of baicalin and oroxyloside in plasma and different tissues of rats after transnasal aerosol inhalation and intravenous injection of Tanreqing

**DOI:** 10.3389/fphar.2022.951613

**Published:** 2022-08-22

**Authors:** Teng-Fei Chen, Ling Song, Yun-Hang Gao, Han Li, Jian-Liang Li, Hong-Ping Hou, Bo Peng, Hui-Ying Wang, Wen-Hao Cheng, Zu-Guang Ye, Ying-Fei Li, Guang-Ping Zhang

**Affiliations:** ^1^ Institute of Chinese Materia Medica, China Academy of Chinese Medical Science, Beijing, China; ^2^ School of Chinese Pharmacy, Beijing University of Chinese Medicine, Beijing, China

**Keywords:** baicalin, lung, oroxyloside, pharmacokinetics, Tanreqing, transnasal aerosol inhalation

## Abstract

To avoid adverse drug reactions associated with injection, off-label nebulization of Tanreqing (TRQ) injection is often used in China to treat respiratory diseases. However, the aerodynamic properties and lung availability of TRQ aerosols remain largely uninvestigated. This study aimed to investigate the size distribution of TRQ aerosols and to compare the pharmacokinetics and tissue distribution of two compounds from TRQ (baicalin and oroxyloside) after transnasal aerosol inhalation and intravenous administration. Furthermore, this study aimed to evaluate the efficacy of TRQ against lipopolysaccharide-induced lung inflammation. The Dv(50) and transmission of TRQ aerosols were 2.512 μm and 74.867%, respectively. The C_max_ of baicalin and oroxyloside in rat plasma after inhalation was lower than that after intravenous injection. After inhalation, the area under the curve (AUC) of baicalin and oroxyloside in tissues (lung, bronchoalveolar lavage fluid, and trachea) was 7.9–115.3 and 9.5–16.0 times that observed after intravenous administration, respectively. Baicalin and oroxyloside maintained high concentrations 4 h after inhalation, but only 1 h after intravenous injection. The mean lung-to-plasma concentration ratios of baicalin and oroxyloside were 287.6 and 49.9 times higher than with intravenous administration. Inhaled TRQ achieved the same effect against lipopolysaccharide-induced lung inflammation in mice at doses of only 1/16–1/8 of those administered intravenously. The results indicate that TRQ inhalation is a promising alternative to intravenous injections for the treatment of respiratory infection.

## Introduction

Tanreqing (TRQ) injection is a Chinese medicinal preparation derived from five traditional Chinese medicines, namely, *Scutellaria baicalensis* Georgi [Lamiaceae; Scutellariae Radix; Huang Qin in Chinese, HQ; 23.6%], *Selenaretos thibetanus* Cuvier [Ursidae; Ursi Fellis Pulvis; Xiong Dan in Chinese, XD; 3.8%], *Naemorhedus goral* Hardwicke [Bovidae; Caprae Hircus Cornu; Shan Yang Jiao in Chinese, SYJ; 1.9%], *Lonicera japonica* Thunb. [Caprifoliaceae; Lonicerae Japonicae Flos; Jin Yin Hua in Chinese, JYH; 23.6%], and *Forsythia suspensa* (Thunb.) Vahl [Oleaceae; Forsythiae Fructus; Lian Qiao in Chinese, LQ; 47.1%] (Rivera et al., 2014; He et al., 2021). The botanical drugs were extracted by water extraction (HQ, JYH, and LQ) and were purified by alcohol sedimentation, hyperfiltration (HQ), saponification (XD), and acid hydrolysis (SYJ), respectively (Mu, 2011). The chemical composition of TRQ includes flavonoids, iridoids, phenolic acids, amino acids, phenethyl alcohol glycosides, lignans, and steroids (Li C et al., 2019; Xie et al., 2021). TRQ possesses anti-inflammatory, antibacterial, antiviral, antitumor, and other pharmacological effects. Furthermore, TRQ can inhibit inflammatory exudation and interstitial pulmonary edema, reduce inflammatory cell infiltration, and prevent acute inflammatory injury to the alveolar epithelium (Zhong et al., 2010; Liu H et al., 2020; Liu W et al., 2020). TRQ was approved by the China Food and Drug Administration in 2003 and has been widely used clinically in the treatment of acute exacerbations of chronic obstructive pulmonary disease, acute upper respiratory tract infections, pneumonia, and other lung diseases (Li et al., 2010; Wang et al., 2011; Wang et al., 2016). However, its clinical applications are limited by the increasing incidence of adverse reactions, including anaphylactic shock (Wang et al., 2018; Li X X et al., 2019).

Compared to intravenous (IV) administration, inhalation does not cause pain and can increase patients’ comfort and compliance, improving the effectiveness of treatment of respiratory diseases. Aerosol inhalation in traditional Chinese medicine has been widely used to clinically treat respiratory diseases, especially the medicine with heat-clearing and detoxifying effects. Lung and respiratory diseases, such as pneumonia, respiratory infections, tracheitis, and bronchitis, can be treated by single or combined aerosolization (Han et al., 2016), demonstrating that traditional Chinese medicines have great potential to treat respiratory diseases. In recent years, numerous studies have reported that TRQ can be clinically used to treat respiratory diseases by aerosol inhalation. Deng and Lei (2017) retrieved 24 related reports involving 1,348 children in domestic and foreign medical databases using TRQ injection, aerosol inhalation, respiratory infections, and pediatric patients as keywords. The results showed that aerosol inhalation of TRQ was found to be safe and effective, with a total clinical efficacy rate >85% and an extremely low incidence of adverse reactions. Through literature analysis and research, Sun et al. (2013) found that TRQ aerosol inhalation was mainly used to treat and prevent respiratory infections in children and the elderly, with significant curative effects and low adverse reactions.

The clinical aerosolization application of TRQ is beyond that indicated on the package insert. Nonetheless, the particle size of the TRQ nebulized aerosols has not been systematically studied, and the absorption and metabolism of multiple components in the lungs after aerosolization are not clear. Based on the chemical composition of TRQ, seven analytes were selected to conduct a preliminary study on pharmacokinetics (PK) after transnasal aerosol inhalation (TI). These analytes include baicalin (BAI) and oroxyloside (ORO, both from HQ), chlorogenic acid (from JYH), forsythoside D and forsythoside E (both from LQ), and ursodeoxycholic acid and chenodeoxycholic acid (both from XD). The preliminary study showed that BAI, ORO, ursodeoxycholic acid, and chenodeoxycholic acid could be detected in rat plasma after TI of TRQ. Ursodeoxycholic acid and chenodeoxycholic acid are endogenous components and have high blood concentrations. The concentration changes of the two compounds after TI administration were difficult to distinguish from the blank. Therefore, BAI and ORO from HQ were selected as the target compounds in the current study. This study evaluated the particle size distribution of TRQ aerosols generated by a nebulizer. The pharmacokinetic characteristics of BAI and ORO were compared in plasma and tissues, including the lung and trachea after TI and IV administration, and the anti-inflammatory effects were investigated in mice lungs.

## Materials and methods

### Reagents and chemicals

**FIGURE 1:**
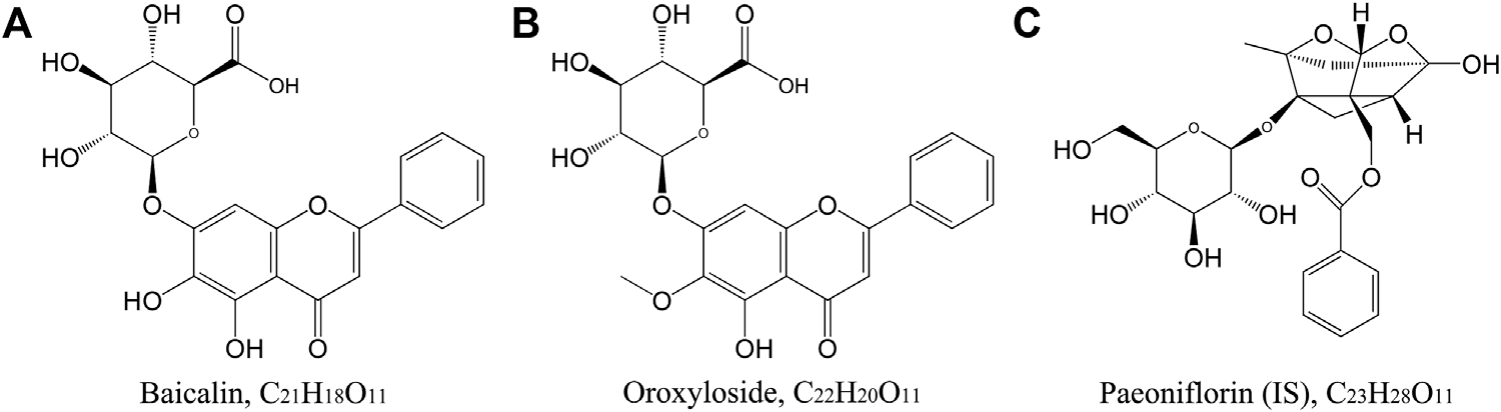
Chemical structure of BAI **(A)**, ORO **(B)**, and PAE (**(C)**, IS).

BAI (CAS No. 21967-41-9, Lot No. 110715-201117, and 91.7% purity) was purchased from the National Institutes for Food and Drug Control (Beijing, China). ORO (CAS No. 36948-76-2, Lot No. SS20190628, and 98.0% purity) was purchased from Chengdu SinoStandards BioTech Co., Ltd. (Chengdu, China). Paeoniflorin [PAE, internal standard (IS), CAS No. 23180-57-6, Lot No. C10833465, and 98.0% purity] was obtained from Shanghai Macklin Biochemical Co. Ltd. (Shanghai, China). The chemical structures of the two analytes and IS are shown in Figure 1. TRQ (Lot No. 2006103) was provided by Shanghai Kaibao Pharmaceutical Co., Ltd. (Shanghai, China). Dexamethasone sodium phosphate (Lot No. 19110802A) was purchased from Guizhou Tiandi Pharmaceutical Co., Ltd. (Guizhou, China). Lipopolysaccharide (LPS, Lot No. 0000114329) was obtained from Sigma-Aldrich Co. (Darmstadt, Germany). Radio immunoprecipitation assay (RIPA) lysis buffer (Lot No. 01408/60527) was purchased from Beijing ComWin Biotech Co., Ltd. (Beijing, China). Mouse interleukin (IL)-6 enzyme-linked immunosorbent assay (ELISA) kit (96T, Lot No. 20211014) was obtained from Beijing Solarbio Science and Technology Co., Ltd. (Beijing, China). Chromatographic methanol and acetonitrile were provided by Merck (Darmstadt, Germany), and chromatographic formic acid was provided by ROE SCIENTIFIC (Newark, United States). Other reagents were commercially available and were analytically and chromatographically pure.

### Animals

The animal procurement and experiments were approved by the Animal Ethics Committee of the Institute of Chinese Materia Medica, China Academy of Chinese Medical Sciences (approval No. 2020B036). Specific pathogen-free (SPF) Sprague–Dawley (SD) rats (200–240 g) and SPF ICR mice (20–24 g) were purchased from the Beijing Vital River Laboratory Animal Technology Co., Ltd. (Beijing, China). All animals were maintained on a 12/12 h light/dark cycle in an environmentally controlled SPF room (temperature 23°C ± 1°C; relative humidity 50% ± 15%). The animals were acclimated to the laboratory for at least 5 days and were unfed 12 h before the experiment, but with free access to water.

### Instrument and analytical conditions

A high-performance liquid chromatography-mass spectrometry (HPLC-MS/MS) system (AB SCIEX, Toronto, Canada) equipped with an ExionLC HPLC tandem with a Qtrap 5500 mass spectrometer was used for analysis and determination. The Shimadzu Shim-pack Velox SP-C18 LC column (100 × 2.1 mm, 2.7 μm) was used as the chromatographic column at a temperature of 40°C. The autosampler temperature was 6°C. After 5 μl injection, mobile phases A (water) and B (methanol: acetonitrile = 1:1) containing 0.05% formic acid were used for elution at a flow rate of 0.3 ml/min according to the following procedure: 0–0.5 min, 95% A; 0.5–0.6 min, 95%–60% A; 0.6–2.0 min, 60% A; 2.0–2.5 min, 60%–5% A; 2.5–3.0 min, 5% A; 3.0–3.1 min, 5%–95% A; 3.1–6.0 min, 95% A.

In the positive ion mode of the electrospray ionization (ESI) source, multiple reaction monitoring scans were used to detect the analytes and IS. The ion spray voltage and curtain gas pressure were maintained at 5500 V and 35 psi, respectively. The collision gas was set at 6 psi, the turbo spray temperature was maintained at 500°C, and the nebulizer (gas 1) and heater gases (gas 2) were set at 45 psi using nitrogen. The precursor/product pairs of BAI, ORO, and IS were *m/z* 447.3→271.0, m*/z* 461.2→284.9, and *m/z* 498.2→179.2, respectively. The declustering potential (DP) of BAI, ORO, and IS was 53.1, 91.9, and 147.0 V, respectively. The collision energy (CE) of BAI, ORO, and IS was 26.9, 26.1, and 23.0 V, respectively. The dwell time and collision exit potential (CXP) for all compounds were 100 ms and 15 V, respectively.

### Blank matrix samples and sample preparation

After anesthesia, blood samples of six rats were collected from the abdominal aorta into heparinized tubes. Plasma was harvested by centrifuging blood samples at 10,000 rpm for 2 min at 4°C. The lung, trachea, and brain were dissected rapidly and harvested. The tissues were rinsed thoroughly, weighed, and processed by homogenization with cold saline in a 1:4, 1:9, and 1:4 (w/v) radio for the lung, trachea, and brain, respectively. Blank plasma and tissue homogenates were stored at −80°C until use.

Biological samples were pre-treated via protein precipitation as follows: 50 μl biological samples were vortex-mixed with 400 μl IS working solution (the plasma samples needed to add 2 μl formic acid first) for 1 min, ultrasound for 2 min, and centrifuged at 14,000 rpm for 5 min at 4°C. The supernatant (400 μl) was centrifuged and concentrated at 1,700 rpm at 47°C until it becomes dry. The residue was redissolved in 100 μl of 70% methanol (0.05% formic acid) followed by a 1 min vortex and a 2 min ultrasound. The supernatant was injected into the HPLC-MS/MS system for analysis after centrifuging at 14,000 rpm for 5 min at 4°C.

For the preparation of working and sample solution, and method validation of HPLC-MS/MS, refer to Supplementary Material.

### Size distribution of TRQ aerosols

TRQ was nebulized using a TurboBoy nebulizer with a red nozzle from PARI Pharma GmbH (Starnberg, Germany). A Spraytec laser diffractometer (Malvern Panalytical Ltd., United Kingdom) was used to evaluate the size distribution of TRQ aerosols generated by the nebulizer at a flow rate of 15 L/min. The experiments were carried out by placing 2 ml of the TRQ solution into the nebulizer cup. The nebulizer cup was then connected to the laser diffractometer and measured. The aerosol transmission volume and particle size distribution were recorded in real-time. The experiment was repeated three times.

### Dose and method of TI

The actual aerosol inhalation dose was calculated using the following equation:

W=C×T×f×TV, (1)

where C is the aerosol concentration at dynamic equilibrium, T is the time of aerosol inhalation, f is the respiratory rate, and TV is the average tidal volume (Zhang et al., 2021). The experimental animals were atomized using the FinePointe oral and nasal exposure system (DSI Buxco, Delaware, United States), which ensured equal concentrations for each animal. The TI groups were nebulized with TRQ for 5, 10, and 20 min. The actual doses calculated according to the formula were 0.03, 0.06, and 0.12 ml/kg for the pharmacokinetic experiment in rats and 0.08, 0.16, and 0.32 ml/kg for the anti-inflammatory experiment in mice.

### Pharmacokinetics of BAI and ORO in rats after TI and IV of TRQ

For the pharmacokinetic experiment, SD rats were randomly divided into four groups (0.03 ml/kg), a low dose group of TI (0.03 ml/kg), a medium dose group of TI (0.06 ml/kg), and a high dose group of TI (0.12 ml/kg), each group consisted of six rats. The animals in each group underwent a jugular vein catheterization the day before the experiment. They were unfed overnight but had free access to water. For the IV group, rats were intravenously injected with TRQ through the tail vein. For the three TI groups, rats were placed in the FinePointe oral and nasal exposure system. A TurboBoy N nebulizer with a red nozzle was used to nebulize TRQ for 5, 10, and 20 min. A photometer was used to record aerosol concentrations at dynamic equilibrium. Before administration and 0.083, 0.167, 0.333, 0.5, 0.75, 1, 2, 4, 6, 8, 10, and 24 h after administration, approximately 0.15 ml of blood was collected from the jugular vein into a 1.5 ml centrifuge tube with heparin anticoagulation followed by centrifuging at 10,000 rpm for 2 min at 4°C. After blood collection at each treatment time, an equal volume of 0.9% saline was added through the jugular vein catheterization to the rat. The 2 μl of formic acid was added to a 50 μl plasma sample and stored at −80°C until analysis. All rats were euthanized with carbon dioxide after sample collection.

### Tissue distribution of BAI and ORO in rats after TI and IV of TRQ

For the tissue distribution experiment, 60 SD rats were randomly divided into the IV group (0.12 ml/kg, intravenously injected through the tail vein) and the TI group (0.12 ml/kg, transnasal aerosol inhalation for 20 min). The inhalation system was the same as that used in the pharmacokinetic experiments. Six rats for each treatment time were anesthetized at 0.083, 0.333, 1, 4, and 8 h after administration, respectively. Blood samples were collected from the abdominal aorta into heparinized tubes. The right lung was clamped, and the left lung was lavaged with 1.5 ml cold saline twice to collect bronchoalveolar lavage fluid (BALF). Subsequently, the right lung, trachea, and brain were dissected rapidly and harvested, and homogenized according to “2.4.”

### Anti-inflammatory effect of TRQ in mice after TI and IV

Seventy male ICR mice were randomized and divided into the normal, model, positive, IV, and low-, medium-, and high-dose groups of TI. There were 10 mice in each group. Except for the normal group, mice in the other six groups were placed in an inhalogic NIES inhalation tower (Melton Lab, Shanghai, China). A 4 mg/ml LPS solution was nebulized for 30 min using a TurboBoy N nebulizer with a red nozzle for modeling. The first intervention was immediately initiated in each group after modeling, and the second intervention was initiated 15 h thereafter. The normal and model groups were nebulized with saline for 20 min. The positive and IV groups were injected with 5.2 mg/kg dexamethasone sodium phosphate and 2.6 ml/kg TRQ (according to the ratio of body surface area of humans and mice, which was calculated from the clinical dose of TRQ, which was 0.29 ml/kg) through the tail vein, respectively. The TI groups were nebulized with TRQ for 5, 10, and 20 min through the inhalation tower. One hour after the last administration, mice in each group were sacrificed. The lungs were dissected rapidly and harvested, and rinsed thoroughly with cold saline. Subsequently, 100 mg of lung tissue was weighed and homogenized in an ice bath after adding 20× (by weight) RIPA lysis buffer. The homogenate was centrifuged at 12,000 rpm for 15 min at 4°C. The level of IL-6 in the lung homogenate was determined by using a mouse IL-6 ELISA kit with a Spectramax i3x reader (Molecular Devices, United States) at 450 nm.

### Data analysis

Data acquisition and analysis for the concentration of BAI and ORO were performed using Analyst 1.7 software (AB SCIEX, Toronto, Canada). Plasma and tissue homogenate concentrations of BAI and ORO were determined using the calibration curve of each analysis batch. Pharmacokinetic parameters were calculated using a noncompartment model with the MaS Studio 1.5.2.14 stable software (Shanghai BioGuider Medicinal Technology Co., Ltd., Shanghai, China). C_max_ and T_max_ were obtained directly from the experimental process.

Data were expressed as mean ± standard deviation (SD), with relative standard deviation (RSD). Multiple data sets were subjected to independent *t*-tests. *p* < 0.05 was considered to be statistically significant.

## Results

### Method optimization and validation

**FIGURE 2:**
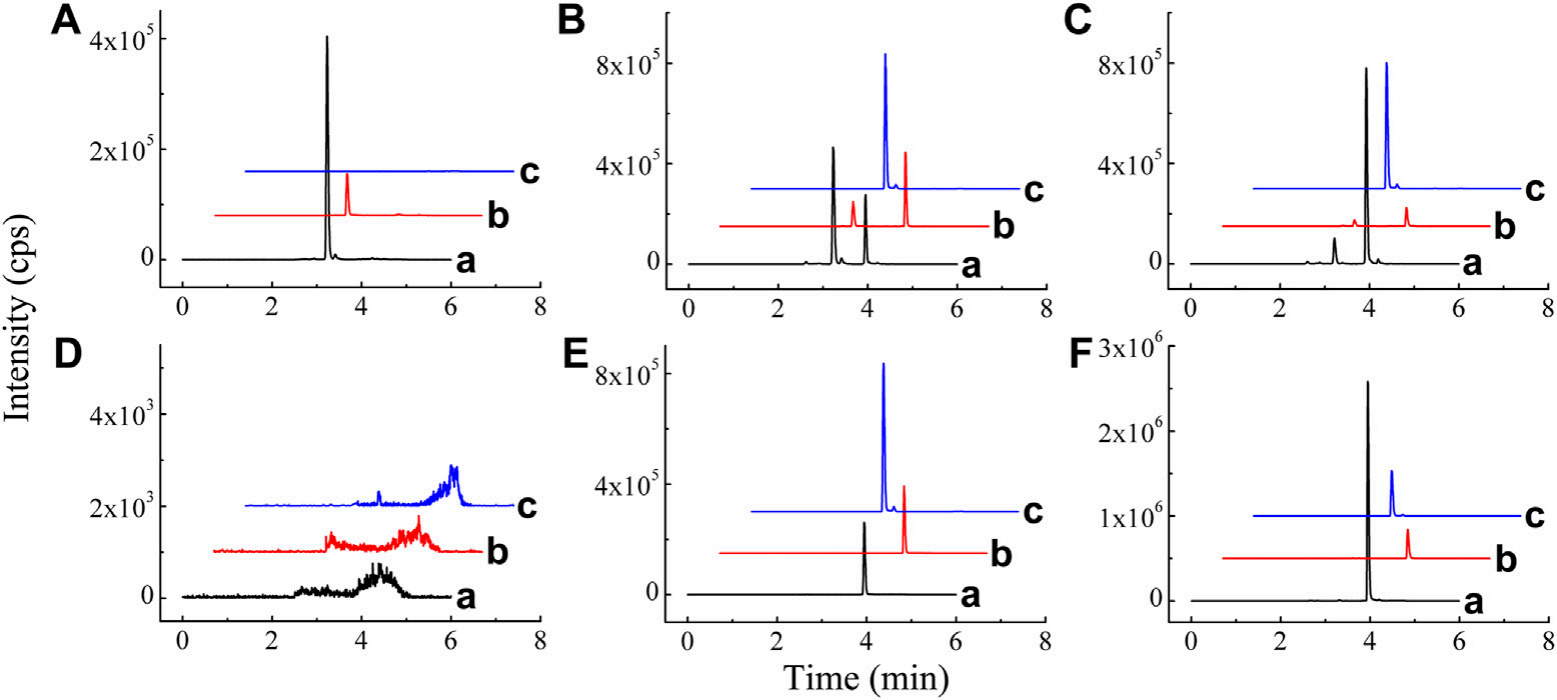
Chromatograms of BAI **(A)**, ORO **(B)**, and IS **(C)** in blank plasma **(A)**; blank plasma spiked with BAI, ORO, and IS **(B)**; plasma sample 20 min after TI of TRQ **(C)**; blank lung homogenate sample **(D)**; blank lung homogenate spiked with BAI, ORO, and IS **(E)**; and lung homogenate sample 10 min after TI of TRQ **(F)**.

Mass spectrometry parameters of BAI, ORO, and PAE (IS) were optimized by using 100 ng/ml methanol solution of the three compounds. In the positive ion mode, the two analytes and IS were more sensitive, which made them suitable for detection in the ESI (+) mode. In ESI (+) mode, DP, CE, CXP, gas 1, gas 2, spray voltage, and curtain gas were optimized to determine the precursor ions and product ions of the three compounds. The optimized chromatographic column was a Shimadzu Shim-pack Velox SP-C18 LC column. The chromatographic separation was performed using water-methanol-acetonitrile containing 0.05% formic acid as the mobile phase system. Representative chromatograms of BAI, ORO, and IS in plasma and lung homogenates are shown in Figure 2. Under the optimized analysis conditions, the retention times of BAI, ORO, and IS were 3.93, 4.13, and 2.99 min, respectively. There were no endogenous and exogenous peaks for the same retention time of analytes and IS in the blank plasma (Figure 2A) and lung homogenate samples (Figure 2D). These results indicated that this method had good specificity and selectivity. This analytical method revealed BAI and ORO in plasma and homogenate samples had a good linear relationship. The linear range of BAI and ORO in the two matrices using 1/X^2^ weighting was 2–200 ng/ml and 1–100 ng/ml, respectively. The lower limit of quantification (LLOQ) of BAI and ORO in the two matrices was 2 ng/ml and 1 ng/ml, respectively. The intra-day and inter-day precision (RSD) and accuracy (RE) in plasma and lung homogenate samples of BAI ranged from 0.47% to 6.25% and 92.23%–114.4%, respectively. The intra-day and inter-day precision and accuracy in plasma and lung homogenate samples of ORO ranged from 0.34% to 7.63% and 90.41%–107.50%, respectively. The dilution procedure showed excellent precision (RSD < 3.74%) and accuracy (RE: 95.80–103.85%). The RSD of relative matrix effects for all analytes ranged from 1.38% to 13.07%, and the average extraction recovery was 37.81%–48.14%. The RSD values ranged from 0.79% to 10.42%, and the deviation values ranged from –13.53% to 6.78%, indicating that BAI and ORO were stable in the autosampler at 6°C for 24 h, at room temperature (25°C) for 1 h, after three freeze/thaw cycles from –80°C to room temperature, and in long-term storage at −80°C for 30 days in plasma and lung samples from rats. The detailed results of the method validation are shown in Supplementary Tables S1–S5.

### TRQ aerosol characteristics

**FIGURE 3:**
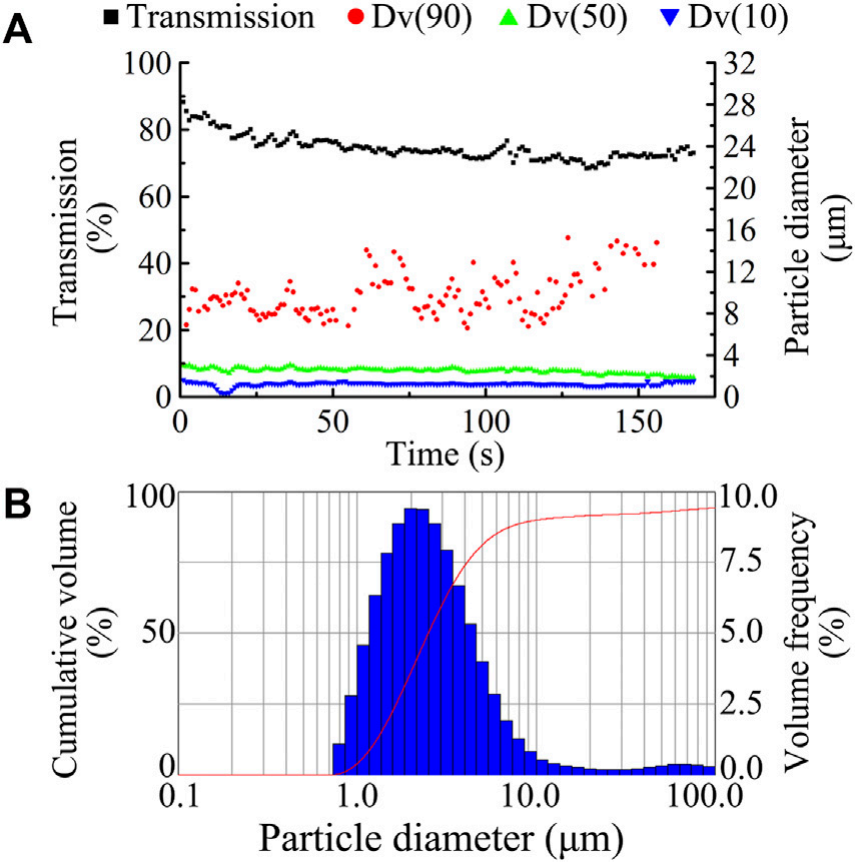
Dynamic changes in aerosol size and transmission (**(A)**, ■ transmission, ● Dv90, ▲ Dv50, and ▼ Dv10) and typical aerosol size distribution **(B)** by using a red nozzle of PARI TurboBoy N nebulizer for the nebulization of TRQ.

The Spraytec laser diffraction system (Malvern) was used to study changes in particle size and percentage transmission of TRQ aerosols nebulized by the PARI TurboBoy N nebulizer with a red nozzle over time, where Dv(10) and Dv(50) remained stable for 3 min, as shown in Figure 3A. The results showed that the red nozzle nebulizer produced aerosols with a uniform particle size (Figure 3B). The mean Dv(50) of the aerosols was 2.512 μm, and the percentage transmittance was 74.867% (Table 1).

**TABLE 1:** Particle size distribution of TRQ aerosols after nebulization using the PARI TurboBoy N nebulizer with red nozzle (*n* = 3).

Spraytec testing	Mean ± SD
Dv (50) (μm)	2.512 ± 0.017
Transmission (%)	74.867 ± 0.551
Span	3.504 ± 0.247
Nebulized time (s)	177.333 ± 9.452

### Pharmacokinetics study of BAI and ORO in rats

**FIGURE 4:**
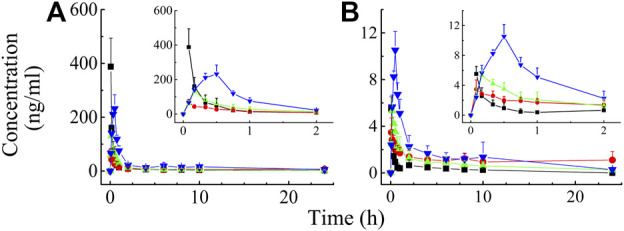
Mean rat plasma concentration-time profiles of BAI **(A)** and ORO **(B)** after IV injection at the dose of 0.03 ml/kg (—■—) and TI at the dose of 0.03 (—●—), 0.06 (—▲—), and 0.12 (—▼—) ml/kg of TRQ (*n* = 6). Bars represent standard deviation.

Validated analytical methods were used to determine the rat plasma concentration of BAI and ORO after IV (0.03 ml/kg) and TI (0.03, 0.06, and 0.12 ml/kg) of TRQ. The plasma concentrations of BAI and ORO at different times after administration are shown in Supplementary Tables S6, S7, respectively. The mean plasma concentration-time profiles are shown in Figure 4.

**FIGURE 5:**
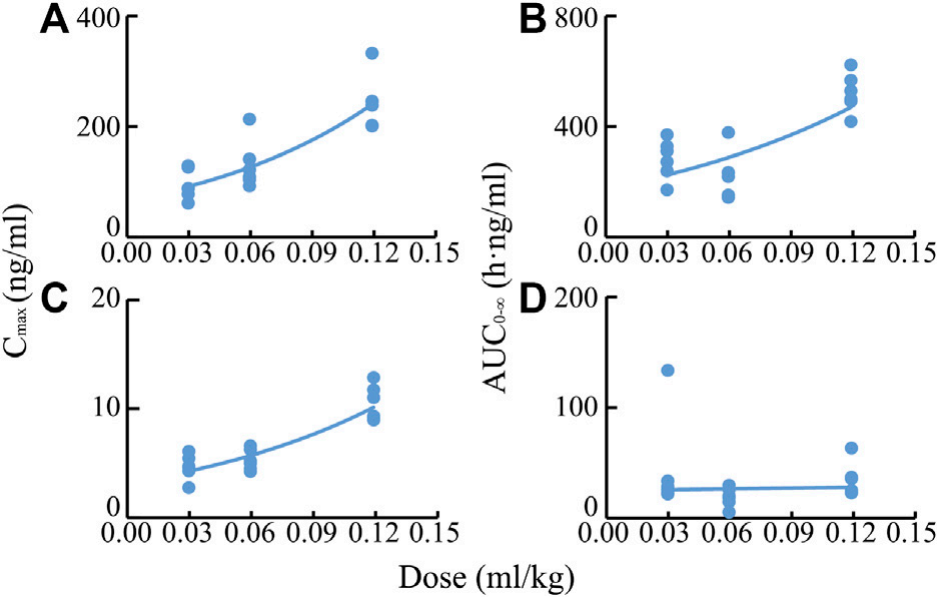
Correlations of C_max_ values of BAI **(A)** and ORO **(C)**, and AUC_0-∞_ values of BAI **(B)** and ORO **(D)** in plasma with TI doses of TRQ (0.03, 0.06, and 0.12 ml/kg).

After IV of TRQ, BAI and ORO were rapidly distributed in 20 min and slowly eliminated (Figure 4). After low, medium, and high dose TI of TRQ, BAI, and ORO were rapidly absorbed. At low, medium, and high doses, BAI and ORO reached C_max_ at 5, 10, and 30 min and were slowly eliminated (Figure 4). Plasma pharmacokinetic parameters of BAI and ORO were calculated using the noncompartment model and are shown in Table 2. After IV injection, T_1/2_ of BAI and ORO was 4.66 h and 4.06 h, and the mean residence time (MRT_0-t_) was 4.51 h and 3.03 h, respectively. The T_1/2_ of BAI (5.17–7.98 h) and ORO (4.89–9.16 h), and the MRT_0-t_ of BAI (6.20–8.12 h) and ORO (5.69–6.73 h) after TI administration were longer than after IV administration. The C_max_ of BAI (69.817, 130.466, and 244.107 ng/ml), and C_max_ of ORO (3.512, 5.318, and 10.527 ng/ml) in plasma after low, medium, and high doses TI of TRQ increased with increasing dose (Figures 5A,C). The mean area under the curve (AUC_0-∞_) of BAI (226.565 h·ng/ml) after medium dose TI of TRQ was slightly lower than that after low dose TI (257.171 h·ng/ml), but the AUC_0-∞_ showed an increasing trend with the increase of TI doses in the range of 0.03-0.12 ml/kg (Figure 5B). The mean AUC_0-∞_ of ORO (25.680, 19.217, and 34.748 h·ng/ml) in plasma after TI did not increase with increasing dose (Figure 5D). The linear relationship between pharmacokinetic parameters and dose was evaluated using confidence intervals (Wang et al., 2008). The correlation between C_max_ of BAI and ORO with doses of TI was positive, but the linear relationship was uncertain. The AUC_0-∞_ of BAI was positively correlated with doses of TI, but the relationship was nonlinear. There was no clear correlation between AUC_0-∞_ of ORO with increasing doses of TI (Table 3).

**TABLE 2:** PK parameters of BAI and ORO in rat plasma after IV at the dose of 0.03 ml/kg and TI at the dose of 0.03, 0.06, and 0.12 ml/kg of TRQ (*n* = 6, Mean ± SD).

Analytes	PK parameters	IV (ml/kg)	TI (ml/kg)		
		0.03	0.03	0.06	0.12
BAI	T_1/2_ (h)	4.662 ± 0.801	7.978 ± 3.567	5.170 ± 0.657	5.492 ± 0.714
	T_max_ (h)	——	0.083 ± 0.000	0.167 ± 0.00	0.444 ± 0.086
	C_max_ (ng/ml)	387.821 ± 106.256	69.817 ± 14.582	130.466 ± 43.830	244.107 ± 47.932
	AUC_0-t_ (h·ng/mL)	230.879 ± 22.456	167.610 ± 57.845	203.747 ± 74.149	475.421 ± 70.913
	AUC_0-∞_ (h·ng/mL)	251.293 ± 19.107	257.171 ± 98.169	226.565 ± 84.421	521.204 ± 70.184
	MRT_0-t_ (h)	4.510 ± 1.271	8.118 ± 3.613	6.228 ± 0.857	6.197 ± 0.451
	MRT_0-∞_ (h)	6.638 ± 2.552	15.992 ± 7.556	8.683 ± 1.461	8.493 ± 1.480
ORO	T_1/2_ (h)	4.057 ± 2.788	9.164 ± 4.043	5.441 ± 1.820	4.886 ± 1.280
	T_max_ (h)	——	0.102 ± 0.033	0.176 ± 0.00	0.500 ± 0.000
	C_max_ (ng/ml)	5.513 ± 1.016	3.512 ± 1.223	5.318 ± 0.937	10.527 ± 1.603
	AUC_0-t_ (h·ng/mL)	5.490 ± 1.307	23.287 ± 10.882	17.172 ± 7.528	32.414 ± 15.338
	AUC_0-∞_ (h·ng/mL)	7.382 ± 2.864	25.680 ± 3.547	19.217 ± 8.453	34.748 ± 15.386
	MRT_0-t_ (h)	3.031 ± 1.089	6.732 ± 2.980	6.310 ± 1.956	5.694 ± 1.613
	MRT_0-∞_ (h)	6.001 ± 5.197	14.619 ± 6.077	8.767 ± 3.040	7.299 ± 1.943

**TABLE 3:** Relationship between dose of TRQ and system exposure levels (C_max_ and AUC_0-∞_) of BAI and ORO in rats after TI (*n* = 6).

Analytes	PK parameters	*r* ^2^	*p value*	Slope (95%CI)	Conclusion^a^
BAI	C_max_	0.709	1.170 × 10^−5^	0.694 (0.458–0.929)	Positive correlation with dose. Uncertainty of linear relationship
	AUC_0-∞_	0.692	1.868 × 10^−5^	0.600 (0.388–0.812)	Positive correlation with dose. Nonlinear correlation
ORO	C_max_	0.341	0.011	0.459 (0.121–0.797)	Positive correlation with dose. Uncertainty of linear relationship
	AUC_0-∞_	0.002	0.864	–0.047 (–0.617–0.523)	No clear correlation with dose

a Dose, C_max_, and AUC_0-∞_ were logarithmic for regression analysis. If the 95% confidence interval (95% CI) of the slope of C_max_ and AUC_0-∞_ was completely within the range of judgment interval (C_max_ 0.74–1.26, AUC_0-∞_ 0.84–1.16), C_max_ and AUC_0-∞_ were linearly correlated within the range of dose (0.03–0.12 ml/kg). If the 95% CI fell completely outside the range of the judgment interval, the correlation was nonlinear. If the 95% CI overlapped with the judgment interval, the linear relationship was uncertain.

### Tissue distribution study of BAI and ORO in rats

**FIGURE 6:**
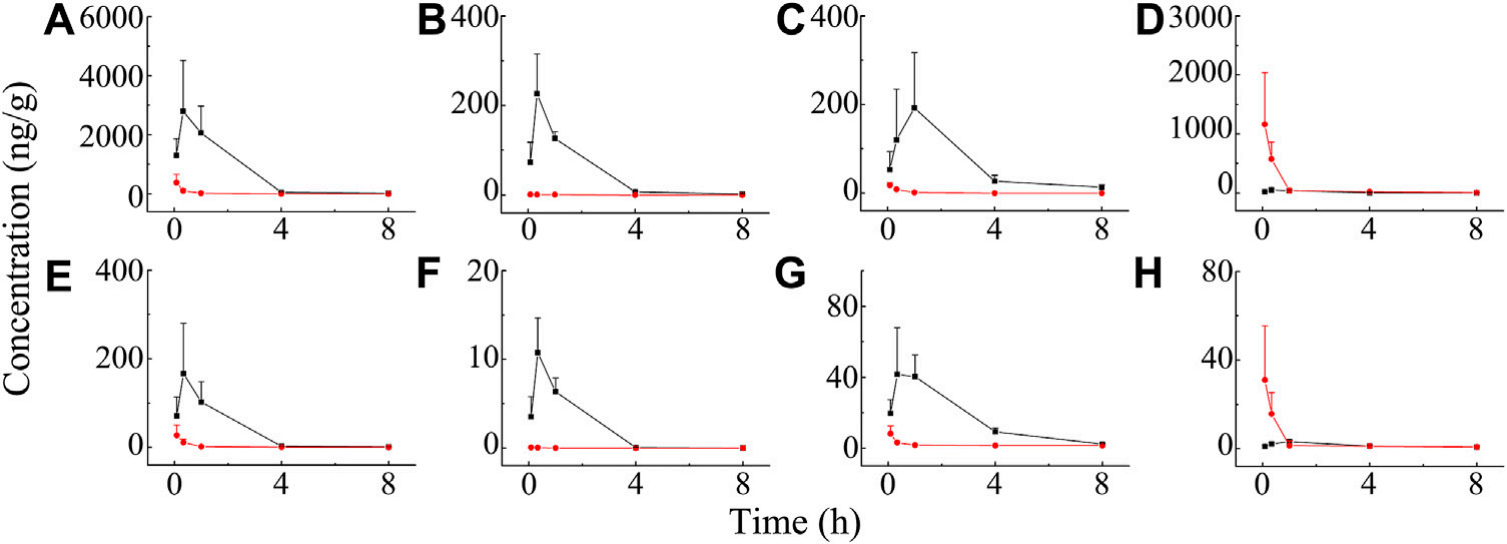
Mean concentration-time profiles of BAI in rat lung **(A)**, BALF **(B)**, trachea **(C)**, plasma **(D)**, and ORO in rat lung **(E)**; BALF **(F)**, trachea **(G)**, and plasma **(H)** after TI (—■—) and IV (—●—) of TRQ both at the dose of 0.12 ml/kg (*n* = 6). Bars represent standard deviation.

The distribution of BAI and ORO in rat lung tissue, BALF, trachea, plasma, and brain after TI and IV administration of TRQ (0.12 ml/kg) were studied. Concentrations of BAI and ORO for biological samples at different sampling times are shown in Supplementary Tables S8, S9, respectively. The mean concentration-time profiles of BAI and ORO in the lung, BALF, trachea, and plasma are shown in Figure 6, and those of the brain are shown in Supplementary Figure S1. The exposure of BAI and ORO in rat tissue and plasma after TI and IV administration of TRQ is listed in Table 4. The pharmacokinetic parameters of BAI and ORO in the lung, BALF, trachea, plasma, and brain, calculated by noncompartmental analysis, are listed in Supplementary Table S10.

**TABLE 4:** Exposure of BAI and ORO in rat lung, BALF, trachea, brain, and plasma after TI and IV of TRQ both at the dose of 0.12 ml/kg (*n* = 6).

Analytes	Biological matrix	PK parameters	TI (0.12 ml/kg)^a^	IV (0.12 ml/kg)
BAI	Lung	C_max_ (ng/ml)	2796.486 (7.379) [52.468]	378.988 [0.326]
		AUC_0-t_ (h·ng/ml)	5502.070 (29.230) [37.142]	188.232 [0.296]
		AUC_0-∞_ (h·ng/ml)	5532.702 (27.970) [32.257]	197.806 [0.303]
	BALF	C_max_ (ng/ml)	226.175 (177.300) [4.243]	1.276 [0.001]
		AUC_0-t_ (h·ng/ml)	375.607 (115.318) [2.536]	3.257 [0.005]
		AUC_0-∞_ (h·ng/ml)	378.552 (91.341) [2.207]	4.144 [0.006]
	Trachea	C_max_ (ng/ml)	192.134 (10.876) [3.605]	17.666 [0.015]
		AUC_0-t_ (h·ng/ml)	535.834 (73.062) [3.617]	7.334 [0.012]
		AUC_0-∞_ (h·ng/ml)	571.582 (72.450) [3.332]	7.889 [0.012]
	Brain	C_max_ (ng/ml)	7.928 (1.078) [0.149]	7.353 [0.006]
		AUC_0-t_ (h·ng/ml)	27.86 (1.202) [0.188]	23.187 [0.037]
		AUC_0-∞_ (h·ng/ml)	57.383 (1.025) [0.335]	55.995 [0.086]
	Plasma	C_max_ (ng/ml)	53.299 (0.046)	1163.122
		AUC_0-t_ (h·ng/ml)	148.135 (0.233)	635.230
		AUC_0-∞_ (h·ng/ml)	171.518 (0.262)	653.895
ORO	Lung	C_max_ (ng/ml)	166.717 (6.112) [51.332]	27.278 [0.879]
		AUC_0-t_ (h·ng/ml)	286.940 (16.970) [22.426]	16.908 [0.821]
		AUC_0-∞_ (h·ng/ml)	288.773 (15.771) [17.345]	18.310 [0.807]
	BALF	C_max_ (ng/ml)	10.763 (166.224) [3.314]	0.065 [0.002]
		AUC_0-t_ (h·ng/ml)	17.270 (1060.761) [1.35]	0.016 [0.001]
		AUC_0-∞_ (h·ng/ml)	17.306 (1062.989) [1.04]	0.016 [0.001]
	Trachea	C_max_ (ng/ml)	41.609 (5.103) [12.811]	8.154 [0.263]
		AUC_0-t_ (h·ng/ml)	133.312 (9.552) [10.419]	13.956 [0.678]
		AUC_0-∞_ (h·ng/ml)	139.142 (5.720) [8.358]	24.327 [1.072]
	Brain	C_max_ (ng/ml)	0.641 (0.703) [0.197]	0.912 [0.029]
		AUC_0-t_ (h·ng/ml)	3.472 (0.937) [0.271]	3.704 [0.180]
		AUC_0-∞_ (h·ng/ml)	8.627 (1.604) [0.518]	5.379 [0.237]
	Plasma	C_max_ (ng/ml)	3.248 (0.105)	31.045
		AUC_0-t_ (h·ng/ml)	12.795 (0.622)	20.587
		AUC_0-∞_ (h·ng/ml)	16.648 (0.734)	22.692

a The values in round brackets “( )” are the ratios of the PK parameters of TI to IV. The values in square brackets “[ ]” are the ratios of the PK parameters of different tissues to those of plasma.

**FIGURE 7:**
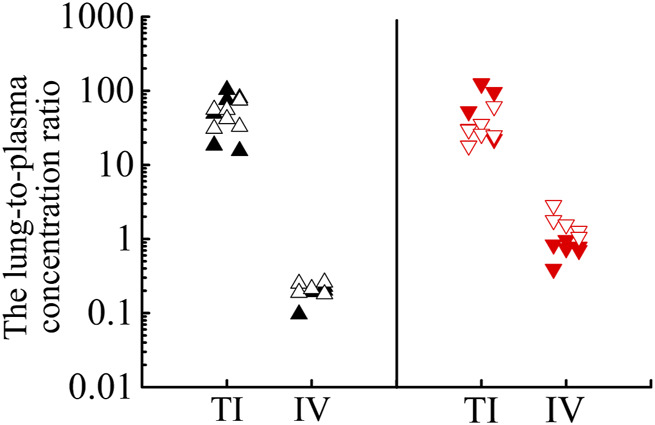
Lung-to-plasma concentration ratios of BAI at 20 min (▲) and 1 h (△), ORO at 20 min (▼) and 1 h (▽) after TI and IV of TRQ at the dose of 0.12 ml/kg.

As shown in Figure 6, after TI of TRQ, BAI, and ORO are rapidly distributed to the lungs, respiratory mucus, and trachea. As the inhalation procedure was carried out, the drug concentration gradually increased. At the end of the inhalation (20 min), the concentration slowly decreased. BAI and ORO in the lungs, BALF, and trachea were maintained at high concentrations for 4 h. However, after 4 h, the concentrations were lower than those at 0.083 h. After inhalation, the concentration of BAI in the lung (Figure 6A) was the highest. Concentrations in BALF (Figure 6B) and trachea (Figure 6C) were almost equivalent and lower than those in lung tissue. The concentration of ORO in the lung (Figure 6E) was the highest, followed by that in the trachea (Figure 6G), and was the lowest in the BALF (Figure 6F). After IV administration of TRQ, BAI, and ORO were quickly distributed to the lungs and trachea with the T_max_ of 5 min (Supplementary Table S10). Subsequently, the concentrations gradually decreased, and their concentrations were significantly lower than those after TI throughout the entire observation period (0–8 h). Only trace amounts of BAI and ORO were detected in BALF after IV administration (Figures 6B,F). It was found that after TI and IV, the two components quickly entered the brain tissue at a low concentration (Supplementary Figure S1; Supplementary Table S10). This study also investigated the permeability of BAI and ORO in the lungs after TI and IV administration of TRQ (Figure 7). The lung-to-plasma drug concentration ratio of BAI ranged from 15.5 to 102.9 at 20 min and 1 h after TI of TRQ, which was approximately 76.4–518.8 times that of the ratio (0.1–1.6) after IV administration at the same dose. The ratio of ORO ranged from 18.1 to 125.4 at 20 min and 1 h after TI of TRQ, which was approximately 6.4–165.8 times that of the ratio (0.40–2.8) after IV administration at the same dose.

### Anti-inflammatory effects of TRQ

**FIGURE 8:**
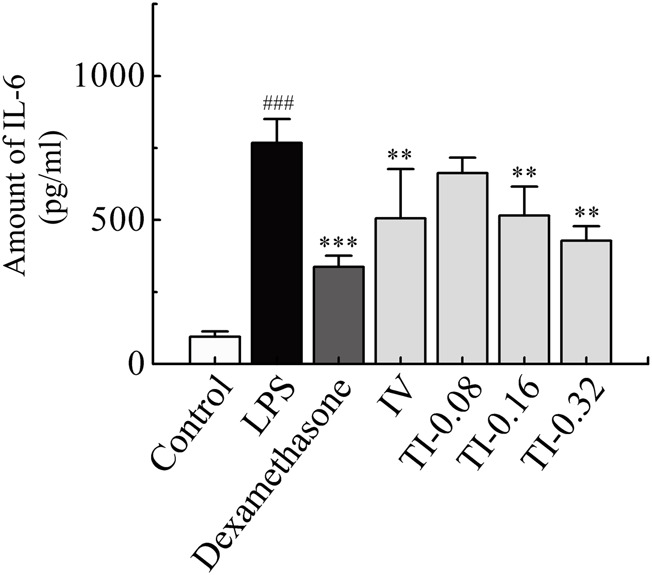
Amount of IL-6 in lung homogenate after TI (5, 10, 20 min) and IV (2.6 ml/kg) of TRQ on LPS-induced mice lung inflammation model (*n* = 10, mean ± SD). ^###^ *p* < 0.001 vs. Control group, ^**^ *p* < 0.01 and ^***^ *p* < 0.001 vs. LPS group.

A mouse pneumonia model was established by aerosol inhalation of LPS at 4 mg/ml. The level of IL-6 in lung homogenates was used as an index to evaluate the model and the anti-inflammatory effect of TRQ (Liu et al., 2016; Rao et al., 2022). Compared to the normal group, the level of IL-6 in the lung homogenate of the model group increased significantly (*p* < 0.001). After administration of dexamethasone, the content of IL-6 in mouse lung homogenate decreased significantly (*p* < 0.001). These results indicated that LPS-induced mice models experienced acute lung injury. Compared to the model group, the content of IL-6 in the IV group (2.6 ml/kg), and in the medium and high dose groups of TI (inhalation for 10 and 20 min, respectively) decreased significantly (*p* < 0.01), as shown in Figure 8.

## Discussion

The TRQ injection extracted and processed from five botanical drugs has been reported to achieve curative effects in the clinical treatment of acute bronchitis, acute pneumonia, chronic obstructive pulmonary disease, and other respiratory infections. In recent years, numerous respiratory diseases have been treated by aerosol inhalation. Although aerosol inhalation administration of TRQ can reduce adverse reactions caused by IV administration, it is still an off-label drug use. For more efficient and safer clinical use, it is necessary to systematically study its inhalable properties, including component analysis, preparation process, pharmacological effects, safety evaluation, pharmacokinetics, and tissue distribution. This study focused on the characteristics of TRQ aerosols, the pharmacokinetics and tissue distribution, and anti-inflammatory effects after TI administration of TRQ.

Based on our preliminary experimental results, the representative flavonoids BAI and ORO in TRQ were selected as the target compounds. BAI, ORO, and IS standard solutions were used to optimize the respective precursor ions and product ions. The analytical conditions of HPLC-MS/MS for BAI and ORO were optimized. This established method has high specificity and sensitivity and can be used for the quantitative analysis of BAI and ORO in biological samples from rats.

Compared to oral administration, TI can avoid the liver first-pass effect and increase drug concentration in the bronchi and alveoli. After delivery to lung tissue, the target organ of respiratory diseases, drug ingredients are absorbed into the blood through the air–blood barrier in the lung alveoli. Subsequently, the active ingredients are distributed to various organs and tissues through blood circulation. The concentration of ingredients entering the systemic circulation after TI is markedly reduced, thus avoiding adverse reactions caused by IV administration.

The size and distribution of aerosol particles are key parameters that influence the nebulization process and the therapeutic effect. Due to the particularity of the respiratory system, aerosol particles of different sizes are deposited at different positions. Moreover, only aerosol particles with a size of 1–5 μm can enter and deposit in the bronchi and alveoli (Chrystyn, 2001; Ibrahim et al., 2015). In addition, commercial nebulizers exhibit significant differences in particle size distribution. Therefore, it is necessary to analyze the particle size and aerodynamic characteristics of aerosols generated by the nebulizer (Loffert et al., 1994; Chang et al., 2020). In this study, the mean Dv(50) of TRQ aerosol was 2.512 μm, which can facilitate the deposition of aerosol particles in the lower respiratory tract, such as the pulmonary alveoli, with good inhalable properties (Reychler et al., 2014).

Systemic adverse reactions are directly related to high systemic exposure, and adverse drug reactions can be reduced with a decrease in C_max_ (China National Medical Products Administration, 2009). After low, medium, and high doses TI of TRQ, the C_max_ of BAI in plasma was lower than that of low dose IV. After low and medium doses TI of TRQ, the C_max_ of ORO in plasma was lower than that of low dose IV. After high-dose TI, the C_max_ of ORO was higher than that of low dose IV. These results may be due to the fact that the drug ingredients after aerosol inhalation need to be absorbed by lung tissues first before entering the systemic blood circulation. It has been reported (Chen et al., 2021) that after intratracheal administration of Shuang-Huang-Lian, the C_max_ of chlorogenic acid, forsythiaside A, and BAI in rats was significantly lower than those following IV administration. After intratracheal administration lasted about 60 min, the plasma concentration decreased, similar to that observed following IV administration. Our previous studies showed that the plasma concentration of the target ingredient after TI was significantly lower than after IV administration (Zhang et al., 2019; Ding et al., 2020).

The local curative effect on respiratory diseases is directly related to the concentration of drugs in the respiratory tissues. Aerosol inhalation can target drug delivery to the lungs. Therefore, administration via inhalation has unique advantages over other routes, such as oral and IV administration, as it avoids the liver first-pass effect and allows the drug to accumulate in the lung or trachea. It compensates for the low concentration of drug components in lung tissue after oral and intravenous administration (Patton and Byron, 2007). After TI of TRQ, the exposure (C_max_ and AUC) of BAI and ORO in rat lung, BALF, and trachea (Table 4) was higher than those achieved after IV at the same dose (BAI, 7.379–177.300 times; ORO, 6.112–1062.989 times). Plasma C_max_ and AUC after TI (Table 4) were lower than those after IV (BAI, 0.046–0.262 times; ORO, 0.105–0.734 times). These results were consistent with the pharmacokinetic results. The lung absorption rate after TI was almost equivalent to that after IV, but there was a significant difference in the distribution *in vivo* after absorption. The high distribution of drug ingredients in the lungs indicated that TI administration has better lung availability than IV administration. Compounds with small molecules can be rapidly absorbed into the lung and their absorption rate depends on the lipophilicity of the drug. The T_max_ of hydrophilic compounds is within 30 min (Patton et al., 2004). The permeability of the drug in lung tissue is expressed as the ratio of the lung drug concentration to the plasma drug concentration. The larger the ratio, the easier it is for the drug to penetrate the lungs. In contrast, the smaller the ratio, the more difficult it is for the drug to enter the lung parenchyma (Goldstein et al., 2002). As shown in Figure 7, BAI and ORO showed good lung permeability. In the anti-inflammatory study, medium and high doses TI of TRQ were approximately 1/16 and 1/8 of those achieved following IV, respectively.

## Conclusion

This study showed that TRQ aerosols produced by the jet nebulizer have good aerodynamic characteristics, which is beneficial for lung deposition. Compared to IV administration, drug exposure in the rat lungs, BALF, and trachea was increased significantly and the local anti-inflammatory efficacy was better after TI administration. In conclusion, according to the analysis of pharmacokinetics, tissue distribution, and anti-inflammatory effects in animals, transnasal aerosol inhalation administration of TRQ has advantages over intravenous administration in the treatment of respiratory diseases. Further clinical studies are needed to confirm the prospects of TRQ inhalation administration.

## Data Availability

The original contributions presented in the study are included in the article/Supplementary Material; further inquiries can be directed to the corresponding authors.

## Abbreviations

AUC, area under the curve; BAI, baicalin; BALF, bronchoalveolar lavage fluid; HQ, huang qin; IS, internal standard; IV, intravenous; JYH, jin yin hua; LPS, lipopolysaccharide; LQ, lian qiao; MRT, mean residence time; ORO, oroxyloside; PAE, paeoniflorin; RSD, relative standard deviation; SYJ, shan yang jiao; TRQ, tanreqing; TI, transnasal aerosol inhalation; XD, xiong dan.
