# Supplementary figures and images for: Pharmacokinetics of baicalin and oroxyloside in plasma and different tissues of rats after transnasal aerosol inhalation and intravenous injection of Tanreqing
Source: Front Pharmacol. 2022 Aug 22;13:951613. doi: 10.3389/fphar.2022.951613 (PMC9442038; doi:10.3389/fphar.2022.951613)

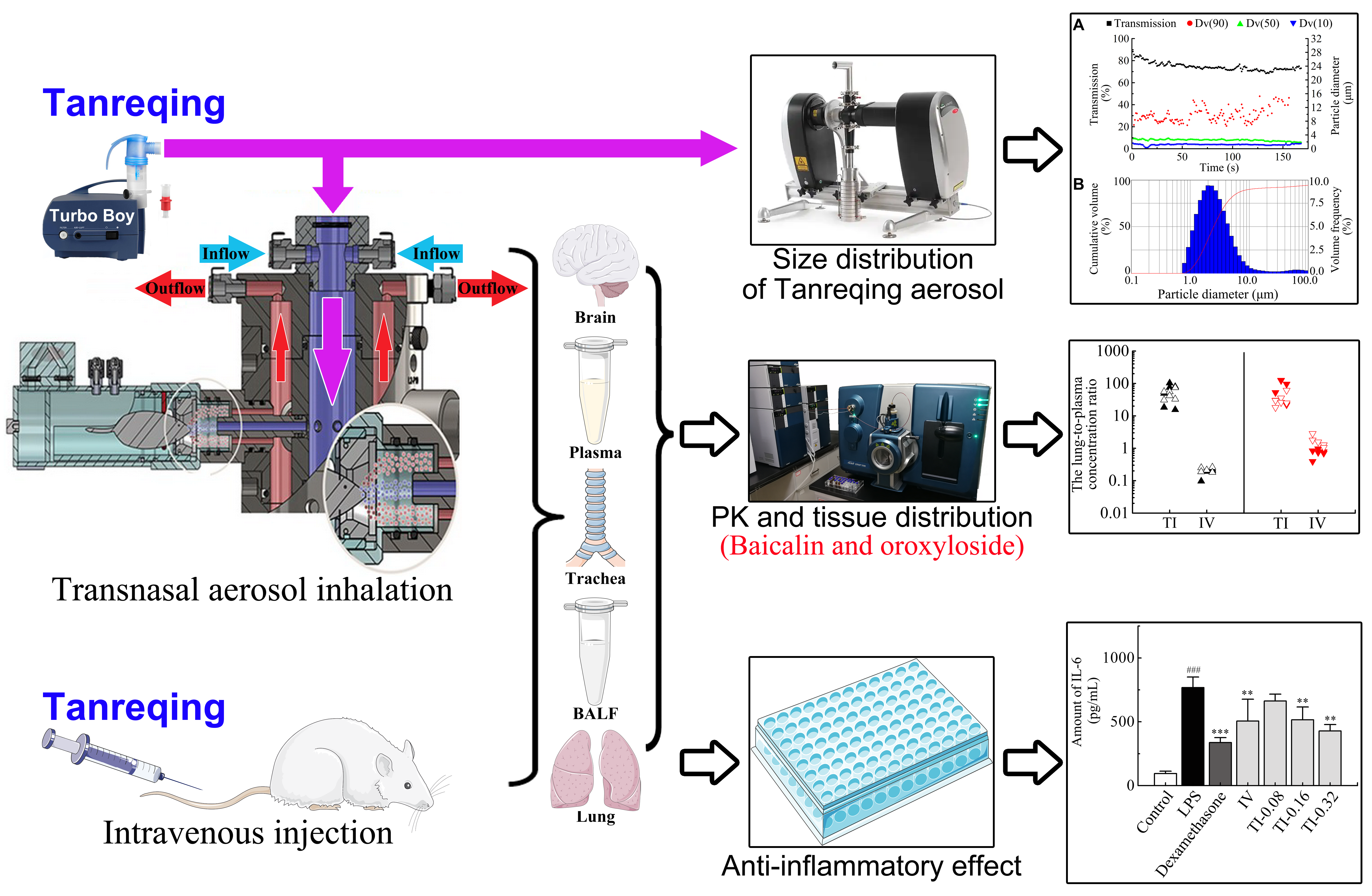

Supplement: Supplementary file 1 [file Image2.TIF]

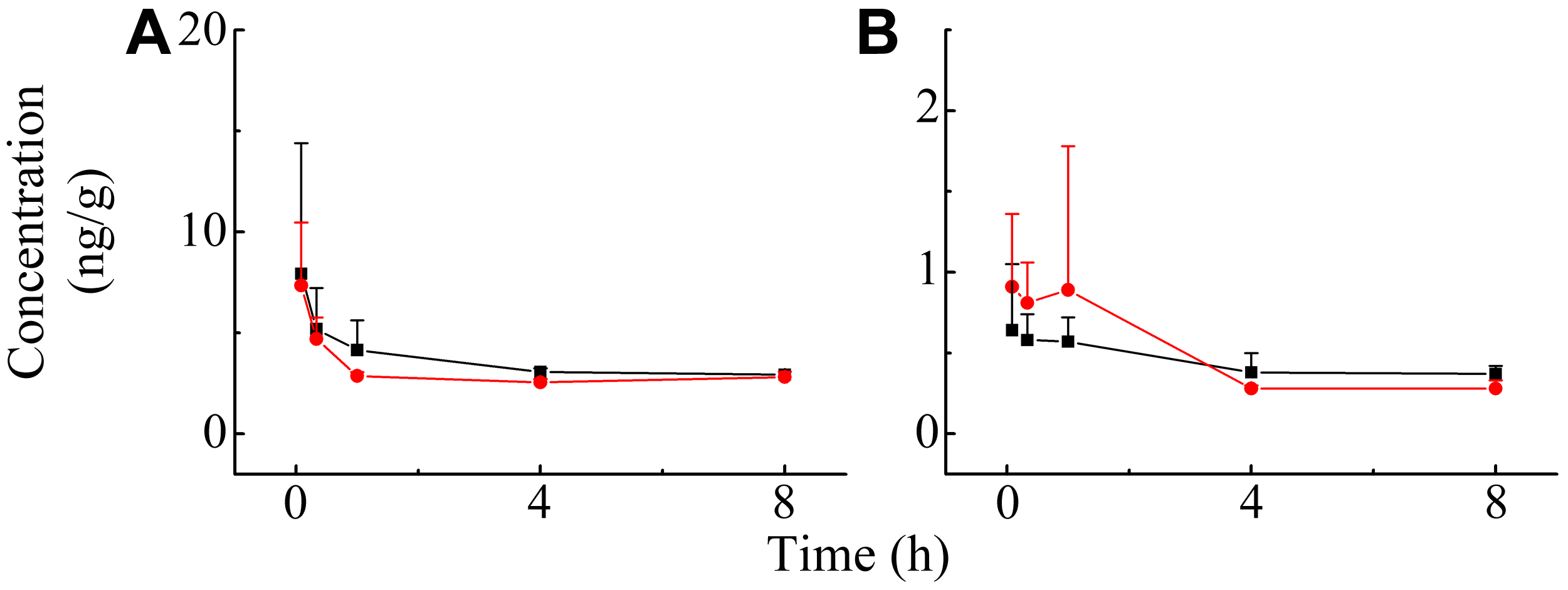

Supplement: Supplementary file 2 [file Image1.TIF]
