# Supplementary material for: Pharmacokinetics of baicalin and oroxyloside in plasma and different tissues of rats after transnasal aerosol inhalation and intravenous injection of Tanreqing
Source: Front Pharmacol. 2022 Aug 22;13:951613. doi: 10.3389/fphar.2022.951613 (PMC9442038; doi:10.3389/fphar.2022.951613)
Supplement: Supplementary file 3 [file DataSheet1.docx]

Supplementary Material for

*Pharmacokinetics of Baicalin and Oroxyloside in Plasma and Different Tissues of Rats After Transnasal Aerosol Inhalation and Intravenous Injection of Tanreqing*

# Working and Sample Solution

Appropriate amounts of BAI and ORO standards were used to accurately weigh and prepare stock solutions with concentration of 1 mg/mL in methanol. The BAI and ORO stock solutions were mixed and diluted with methanol to obtain a series of mixed standard curve working solutions with BAI and ORO concentrations of 20−2000 and 10−1000 ng/mL, respectively, and quality control (QC) working solutions with BAI concentrations of 40, 200 and 1500 ng/mL and ORO concentrations of 20, 100, and 750 ng/mL. An IS stock solution with concentration of 1 mg/mL in methanol was prepared with an appropriate amount of PAE. The IS stock solution was then diluted with acetone containing 0.05% formic acid into an IS working solution with a 15 ng/mL final PAE concentration. All working solutions were stored at −80 °C.

Five microliters of the standard working solution and the low, medium and high QC working solutions were mixed with 45 μL of blank biological sample to prepare standard curve samples and QC samples.

# Method Validation

The developed HPLC-MS/MS method was validated according to the Food and Drug Administration guidelines for the validation of industrial bioanalytical methods. The evaluation indicators included specificity, the lower limit of quantification (LLOQ), linearity, inter- and intra-day precision and accuracy, dilution reliability, matrix effect, extraction recovery, and stability of BAI and ORO in blank plasma and tissue homogenates.

The standard curve was obtained using the weighted least squares method with the concentration of BAI (2, 4, 10, 20, 40, 100, and 200 ng/mL) or ORO (1, 2, 5, 10, 20, 50, and 100 ng/mL) as the horizontal axis and the peak area ratio as the vertical axis. Except for the LLOQ samples with an accuracy between 80% and 120%, the accuracy of the other concentration samples was between 85% and 115%, and the correlation coefficient (*r*) was greater than 0.99. Biological samples with concentrations above the upper limit of quantification (ULOQ) were verified for dilution reliability. Plasma or lung tissue samples with respective concentrations of BAI and ORO (750 and 375 ng/mL) were diluted with blank plasma or lung tissue homogenate five times before processing and analysis. The actual concentration of each sample was determined by multiplying the dilution factor, and both precision and accuracy were in the range of 85−115%. The BAI and ORO stability in different biological samples was investigated under four conditions: processed sample at 6 °C for 24 h; short-term storage at room temperature (25 °C) for 1 h; three freeze/thaw cycles (−80 °C → room temperature); and long-term storage at −80 °C for 30 days by comparing the concentrations of BAI and ORO at low, medium, and high QC levels under different conditions with those of newly prepared samples of the same concentrations. The deviation was within ±15%.

**Table S1.** Linear equations, the linear range for HPLC-MS/MS analysis of BAI and ORO in rat plasma and tissue homogenate.

| Analytes | Biological  matrix | Calibration curves | Linear range  (ng/mL) | Correlation  coefficients (*r*) |
| --- | --- | --- | --- | --- |
| BAI | Plasma | y=0.00941x+0.000948 | 2−200 | 0.9945 |
|  | Lung | y=0.0116x-0.00337 | 2−200 | 0.9980 |
|  | Trachea | y=0.00739x-0.00516 | 2−200 | 0.9938 |
|  | Brain | y=0.0116x-0.00473 | 2−200 | 0.9903 |
|  | Balf | y=0.0139x-0.0000631 | 2−200 | 0.9983 |
| ORO | Plasma | y=0.0286x+0.00605 | 1−100 | 0.9991 |
|  | Lung | y=0.035x-0.00163 | 1−100 | 0.9982 |
|  | Trachea | y=0.0377x-0.005 | 1−100 | 0.9995 |
|  | Brain | y=0.0357x-0.00153 | 1−100 | 0.9996 |
|  | Balf | y=0.0283x+0.00374 | 1−100 | 0.9997 |

**Table S2.** Precision and accuracy of BAI and ORO in rat plasma and lung homogenate.

| Biological  matrix | Analytes | Nominal  concentration  (ng/mL) | Intra-day (n=5) | | Inter-day (n=5×3) | |
| --- | --- | --- | --- | --- | --- | --- |
|  |  |  | Precision  (RSD, %) | Accuracy  (RE, %) | Precision  (RSD, %) | Accuracy  (RE, %) |
| Plasma | BAI | 2 | 3.29 | 114.40 | 6.25 | 108.67 |
|  |  | 4 | 2.25 | 92.23 | 5.80 | 98.49 |
|  |  | 20 | 1.01 | 96.98 | 4.50 | 102.94 |
|  |  | 150 | 0.47 | 99.18 | 3.54 | 103.05 |
|  | ORO | 1 | 2.70 | 95.88 | 3.98 | 96.27 |
|  |  | 2 | 2.63 | 90.41 | 7.63 | 99.97 |
|  |  | 10 | 1.36 | 103.91 | 2.53 | 106.08 |
|  |  | 75 | 0.34 | 101.79 | 2.17 | 101.67 |
| Lung | BAI | 2 | 2.42 | 106.45 | 1.82 | 105.95 |
|  |  | 4 | 1.11 | 101.35 | 1.33 | 101.12 |
|  |  | 20 | 1.44 | 105.25 | 1.15 | 104.42 |
|  |  | 150 | 3.83 | 107.55 | 3.78 | 106.45 |
|  | ORO | 1 | 1.75 | 101.82 | 3.81 | 100.23 |
|  |  | 2 | 2.31 | 107.50 | 2.89 | 104.38 |
|  |  | 10 | 1.01 | 105.36 | 1.54 | 103.87 |
|  |  | 75 | 0.97 | 102.26 | 2.14 | 100.04 |

**Table S3.** Stability of BAI and ORO in rat plasma and lung homogenate (n=5).

| Biological  matrix | Analytes | Nominal  concentration  (ng/mL) | Autosampler | | Room  temperature | | Freeze/thaw | | Long-term | |
| --- | --- | --- | --- | --- | --- | --- | --- | --- | --- | --- |
|  |  |  | RSD  (%) | bias  (%) | RSD  (%) | bias  (%) | RSD  (%) | bias  (%) | RSD  (%) | bias  (%) |
| Plasma | BAI | 4 | 4.32 | 5.33 | 4.21 | –1.26 | 4.69 | 1.07 | 8.35 | –1.90 |
|  |  | 20 | 1.25 | 5.61 | 1.94 | –0.52 | 2.33 | 1.29 | 4.43 | –0.13 |
|  |  | 150 | 1.74 | 6.12 | 2.70 | –0.42 | 2.65 | 2.31 | 10.42 | –6.36 |
|  | ORO | 2 | 3.02 | –8.72 | 3.23 | –5.53 | 1.09 | –1.84 | 7.84 | –3.83 |
|  |  | 10 | 1.98 | –3.99 | 1.71 | –0.73 | 2.34 | 0.29 | 3.07 | –7.62 |
|  |  | 75 | 2.62 | –2.05 | 2.31 | –1.63 | 2.88 | 0.16 | 4.21 | –4.95 |
| Lung | BAI | 4 | 1.38 | –1.14 | 2.59 | 4.26 | 0.95 | 0.42 | 4.67 | –4.59 |
|  |  | 20 | 1.88 | –1.86 | 2.39 | 1.18 | 3.06 | 3.55 | 5.05 | –11.06 |
|  |  | 150 | 4.16 | –3.65 | 5.01 | –4.12 | 2.44 | –1.04 | 6.41 | –13.53 |
|  | ORO | 2 | 0.79 | 2.85 | 2.84 | 6.78 | 1.34 | –2.61 | 2.14 | –2.00 |
|  |  | 10 | 1.24 | –2.38 | 2.12 | 0.70 | 1.48 | 2.44 | 3.98 | –7.33 |
|  |  | 75 | 1.10 | –3.54 | 1.50 | –4.35 | 2.67 | 0.58 | 3.94 | –8.65 |

**Table S4.** Matrix effect and extraction recovery of BAI and ORO in rat plasma and lung homogenate (n=6).

| Biological  matrix | Analytes | Nominalconcentration  (ng/mL) | Matrix effect  (RSD, %) | Extraction recovery  (Mean ± SD, %) |
| --- | --- | --- | --- | --- |
| Plasma | BAI | 4 | 4.19 | 48.14 ± 3.63 |
|  |  | 150 | 3.68 | 45.85 ± 2.09 |
|  | ORO | 2 | 3.96 | 37.81 ± 2.87 |
|  |  | 75 | 7.70 | 46.06 ± 4.57 |
| Lung | BAI | 4 | 1.65 | 43.24 ± 2.25 |
|  |  | 150 | 1.38 | 40.49 ± 3.11 |
|  | ORO | 2 | 8.92 | 41.94 ± 3.52 |
|  |  | 75 | 13.07 | 39.44 ± 2.40 |

**Table S5.** Dilution reliability of BAI and ORO in rat plasma and lung homogenate (Mean ± SD, n=6).

| Biological  matrix | Compounds | Dilution  factor | Concentration (ng/mL) | | Precision  (RSD, %) | Accuracy  (%) |
| --- | --- | --- | --- | --- | --- | --- |
|  |  |  | Nominal | Measured |  |  |
| Plasma | BAI | 5 | 750.00 | 758.60 ± 21.72 | 2.86 | 101.15 |
|  | ORO | 5 | 375.00 | 359.24 ± 4.84 | 1.35 | 95.80 |
| Lung | BAI | 5 | 750.00 | 778.84 ± 29.11 | 3.74 | 103.85 |
|  | ORO | 5 | 375.00 | 363.27 ± 5.47 | 1.51 | 96.87 |

**Table S6.** Plasma concentrations of BAI at different time points after IV and TI of TRQ in rats.

| Dose  (mL/kg) | Time  (h) | Concentration (ng/mL) | | | | | | |
| --- | --- | --- | --- | --- | --- | --- | --- | --- |
|  |  | 1 | 2 | 3 | 4 | 5 | 6 | Mean ± SD |
| IV  (0.03) | 0.083 | 279.95 | 371.41 | 489.33 | 426.77 | 507.36 | 252.03 | 387.81 ± 106.26 |
|  | 0.167 | 260.31 | 139.40 | 153.70 | 114.31 | 126.63 | 164.22 | 159.76 ± 52.43 |
|  | 0.333 | 141.94 | 60.82 | 56.17 | 37.76 | 63.21 | 80.98 | 73.48 ± 36.29 |
|  | 0.5 | 133.06 | 30.23 | 30.18 | 28.69 | 52.28 | 29.58 | 50.67 ± 41.37 |
|  | 0.75 | 49.07 | 15.11 | 17.92 | 9.39 | 19.90 | 15.66 | 21.17 ± 14.12 |
|  | 1 | 26.10 | 10.43 | 10.82 | 10.86 | 14.56 | 7.01 | 13.30 ± 6.71 |
|  | 2 | 7.49 | 5.52 | 10.20 | 12.61 | 10.65 | 5.50 | 8.66 ± 2.94 |
|  | 4 | 5.04 | 4.74 | 4.46 | 7.52 | 5.25 | 11.45 | 6.41 ± 2.70 |
|  | 6 | 3.90 | 4.64 | 3.54 | 6.54 | 3.86 | 8.04 | 5.09 ± 1.81 |
|  | 8 | 4.10 | 4.45 | 4.28 | 4.22 | 5.30 | 6.72 | 4.84 ± 1.01 |
|  | 10 | 3.55 | 2.99 | 3.89 | 3.41 | 4.02 | 4.45 | 3.72 ± 0.51 |
|  | 24 | 2.44 | 5.33* | 3.52 | 1.84 | 1.37 | 2.78 | 2.39 ± 1.41 |
| TI  (0.03) | 0.083 | 80.04 | 77.64 | 88.26 | 49.01 | 62.51 | 61.43 | 69.82 ± 14.58 |
|  | 0.167 | 129.19 | 44.45 | 31.51 | 72.48 | 126.48 | 53.17 | 76.21 ± 42.16 |
|  | 0.333 | 44.42 | 36.13 | 21.10 | 87.90 | 50.82 | 45.76 | 47.69 ± 22.28 |
|  | 0.5 | 17.22 | 25.34 | 21.06 | 78.85 | 44.35 | 28.11 | 35.82 ± 23.05 |
|  | 0.75 | 18.46 | 15.30 | 7.35 | 40.65 | 30.43 | 31.47 | 23.94 ± 12.31 |
|  | 1 | 14.39 | 19.25 | 7.99 | 27.61 | 16.23 | 8.78 | 15.71 ± 7.26 |
|  | 2 | 8.07 | 7.04 | 7.22 | 19.08 | 6.52 | 5.22 | 8.86 ± 5.09 |
|  | 4 | 3.77 | 4.09 | 11.96 | 11.14 | 6.93 | 14.91 | 8.80 ± 4.55 |
|  | 6 | 3.02 | 4.31 | 3.52 | 9.09 | 4.60 | 7.83 | 5.39 ± 2.47 |
|  | 8 | 7.10 | 5.35 | 4.89 | 12.37 | 8.64 | 31.36* | 7.67 ± 3.02 |
|  | 10 | 6.77 | 4.73 | 6.60 | 5.28 | 6.94 | 8.93 | 6.54 ± 1.47 |
|  | 24 | 6.38 | 6.52 | 7.90 | 6.08 | 9.83 | 36.58* | 7.34 ± 1.56 |
| TI  (0.06) | 0.083 | 36.96 | 53.07 | 77.08 | 54.09 | 48.56 | 84.80 | 59.09 ± 18.14 |
|  | 0.167 | 141.16 | 109.33 | 213.32 | 92.75 | 121.91 | 104.33 | 130.47 ± 43.83 |
|  | 0.333 | 62.61 | 85.51 | 62.29 | 86.47 | 90.61 | 75.60 | 77.18 ± 12.43 |
|  | 0.5 | 38.06 | 65.60 | 44.45 | 78.68 | 62.75 | 70.79 | 60.05 ± 15.67 |
|  | 0.75 | 24.89 | 47.23 | 17.55 | 56.24 | 30.28 | 54.57 | 38.46 ± 16.38 |
|  | 1 | 19.58 | 23.18 | 12.4 | 40.69 | 20.07 | 49.61 | 27.59 ± 14.33 |
|  | 2 | 8.27 | 11.53 | 7.76 | 8.22 | 3.99 | 43.77 | 13.92 ± 14.82 |
|  | 4 | 8.60 | 6.80 | 5.79 | 5.27 | 2.40 | 14.12 | 7.16 ± 3.97 |
|  | 6 | 9.47 | 8.20 | 6.17 | 7.45 | 3.26 | 8.63 | 7.20 ± 2.23 |
|  | 8 | 8.86 | 6.46 | 4.34 | 4.46 | 3.55 | 10.50 | 6.36 ± 2.79 |
|  | 10 | 6.59 | 7.02 | 2.62 | 5.58 | 3.49 | 10.12 | 5.90 ± 2.69 |
|  | 24 | 3.61 | 3.03 | 1.61 | 2.93 | 1.73 | 4.87 | 2.96 ± 1.22 |
| TI  (0.12) | 0.083 | 70.56 | 55.31 | 57.77 | 93.97 | 63.84 | 60.79 | 67.04 ± 14.21 |
|  | 0.167 | 160.12 | 91.67 | 170.85 | 169.4 | 132.85 | 117.35 | 140.37 ± 32.00 |
|  | 0.333 | 238.19 | 199.59 | 202.11 | 244.73 | 181.83 | 199.53 | 211.00 ± 24.77 |
|  | 0.5 | 198.09 | 202.36 | 246.00 | 210.89 | 200.78 | 332.58 | 231.78 ± 52.46 |
|  | 0.75 | 97.85 | 128.46 | 117.95 | 107.93 | 117.62 | 140.76 | 118.43 ± 15.06 |
|  | 1 | 66.83 | 112.64 | 66.66 | 62.71 | 63.54 | 78.01 | 75.07 ± 19.21 |
|  | 2 | 22.16 | 24.75 | 16.65 | 19.19 | 19.88 | 29.87 | 22.08 ± 4.70 |
|  | 4 | 27.99 | 11.50 | 4.95 | 16.22 | 6.14 | 11.79 | 13.10 ± 8.37 |
|  | 6 | 13.53 | 11.34 | 13.74 | 43.59 | 14.97 | 15.38 | 18.76 ± 12.25 |
|  | 8 | 18.80 | 11.79 | 15.06 | 11.39 | 8.69 | 19.37 | 14.18 ± 4.30 |
|  | 10 | 13.11 | 12.52 | 11.26 | 22.10 | 14.63 | 22.28 | 15.98 ± 4.93 |
|  | 24 | 6.58 | 6.06 | 7.96 | 6.38 | 3.37 | 3.28 | 5.60 ± 1.88 |

ND: Failed to check out. *: Abnormal value, not calculated.

**Table S7.** Plasma concentrations of ORO at different time points after IV and TI of TRQ in rats.

| Dose  (mL/kg) | Time  (h) | Concentration (ng/mL) | | | | | | |
| --- | --- | --- | --- | --- | --- | --- | --- | --- |
|  |  | 1 | 2 | 3 | 4 | 5 | 6 | Mean ± SD |
| IV  (0.03) | 0.083 | 4.94 | 5.37 | 6.66 | 5.13 | 6.79 | 4.20 | 5.51 ± 1.02 |
|  | 0.167 | 4.52 | 2.47 | 2.59 | 1.39 | 1.64 | 2.77 | 2.56 ± 1.11 |
|  | 0.333 | 2.55 | 1.12 | 1.33 | 0.86 | 1.18 | 1.54 | 1.43 ± 0.60 |
|  | 0.5 | 2.36 | 0.74 | 0.64 | 0.49 | 0.75 | 0.65 | 0.94 ± 0.70 |
|  | 0.75 | 1.17 | 0.47 | 0.37 | 0.1 | 0.54 | 0.08 | 0.45 ± 0.40 |
|  | 1 | 0.74 | 0.32 | 0.25 | 0.4 | 0.41 | 0.19 | 0.38 ± 0.19 |
|  | 2 | 0.52 | 0.47 | 0.6 | 1.54 | 0.61 | 0.19 | 0.65 ± 0.46 |
|  | 4 | 0.84 | 0.21 | 0.61 | 0.44 | 0.25 | 1.14 | 0.58 ± 0.36 |
|  | 6 | 0.62 | 0.28 | 0.41 | 0.31 | 0.17 | 0.42 | 0.37 ± 0.15 |
|  | 8 | 0.21 | 0.26 | 0.36 | 0.32 | ND | 0.89 | 0.41 ± 0.28 |
|  | 10 | 0.39 | 0.09 | 0.18 | 0.09 | ND | 0.54 | 0.26 ± 0.20 |
|  | 24 | ND | 1.81* | ND | ND | ND | ND | —— |
| TI  (0.03) | 0.083 | 3.70 | 4.27 | 5.47 | 2.51 | 2.33 | 2.58 | 3.47 ± 1.24 |
|  | 0.167 | 6.10 | 4.73 | 2.91 | 4.37 | 4.31 | 2.78 | 4.20 ± 1.23 |
|  | 0.333 | 3.85 | 3.31 | 2.11 | 4.63 | 2.52 | 2.36 | 3.13 ± 0.98 |
|  | 0.5 | 1.67 | 2.77 | 1.76 | 3.68 | 2.82 | 1.71 | 2.40 ± 0.82 |
|  | 0.75 | 1.67 | 2.59 | 1.39 | 2.14 | 1.64 | 2.13 | 1.93 ± 0.44 |
|  | 1 | 1.85 | 2.27 | 1.77 | 2.08 | 1.36 | 0.90 | 1.71 ± 0.50 |
|  | 2 | 1.88 | 1.48 | 1.23 | 2.06 | 0.92 | 0.67 | 1.37 ± 0.54 |
|  | 4 | 0.92 | 1.32 | 1.29 | 0.97 | 0.69 | 1.58 | 1.13 ± 0.32 |
|  | 6 | 1.26 | 1.27 | 1.09 | 0.80 | 0.51 | 1.39 | 1.05 ± 0.33 |
|  | 8 | 1.78 | 1.00 | 0.90 | 1.15 | 0.67 | 1.84 | 1.22 ± 0.48 |
|  | 10 | 3.09* | 1.09 | 1.03 | 0.88 | 0.86 | 0.89 | 0.95 ± 0.10 |
|  | 24 | 1.75 | 2.06* | 2.76* | 0.45 | 0.51 | 1.73* | 0.90 ± 0.73 |
| TI  (0.06) | 0.083 | 1.77 | 2.24 | 4.78 | 2.08 | 2.14 | 2.20 | 2.54 ± 1.11 |
|  | 0.167 | 6.26 | 5.23 | 6.61 | 5.02 | 4.53 | 4.26 | 5.32 ± 0.94 |
|  | 0.333 | 3.62 | 4.86 | 4.39 | 4.88 | 3.85 | 3.73 | 4.22 ± 0.57 |
|  | 0.5 | 2.68 | 3.62 | 3.66 | 4.71 | 2.36 | 4.03 | 3.51 ± 0.87 |
|  | 0.75 | 1.80 | 2.86 | 1.84 | 3.11 | 1.19 | 3.05 | 2.31 ± 0.80 |
|  | 1 | 1.69 | 1.86 | 1.22 | 2.70 | 1.15 | 3.84 | 2.08 ± 1.03 |
|  | 2 | 1.01 | 1.09 | 0.92 | 1.02 | 0.40 | 3.08 | 1.26 ± 0.93 |
|  | 4 | 1.10 | 0.93 | 0.70 | 0.85 | 0.29 | 1.63 | 0.92 ± 0.44 |
|  | 6 | 1.68 | 1.16 | 0.64 | 0.75 | 0.15 | 1.02 | 0.90 ± 0.52 |
|  | 8 | 1.20 | 0.78 | 0.49 | 0.56 | 0.25 | 0.90 | 0.70 ± 0.33 |
|  | 10 | 0.98 | 0.72 | 0.37 | 0.42 | 0.17 | 0.99 | 0.61 ± 0.34 |
|  | 24 | 0.30 | 0.10 | 0.20 | 0.38 | ND | 0.31 | 0.26 ± 0.11 |
| TI  (0.12) | 0.083 | 2.26 | 2.29 | 2.38 | 3.13 | 1.97 | 2.27 | 2.39 ± 0.39 |
|  | 0.167 | 5.05 | 5.21 | 7.42 | 6.26 | 5.31 | 4.73 | 5.67 ± 1.00 |
|  | 0.333 | 8.76 | 7.98 | 8.41 | 8.59 | 7.37 | 8.32 | 8.24 ± 0.50 |
|  | 0.5 | 9.20 | 9.33 | 11.03 | 8.98 | 11.76 | 12.87 | 10.53 ± 1.60 |
|  | 0.75 | 5.47 | 7.37 | 6.19 | 6.44 | 6.56 | 8.34 | 6.73 ± 1.00 |
|  | 1 | 4.44 | 7.25 | 5.10 | 4.11 | 4.06 | 5.62 | 5.1 ± 1.21 |
|  | 2 | 2.12 | 2.60 | 1.44 | 1.77 | 1.54 | 4.01 | 2.25 ± 0.96 |
|  | 4 | 1.94 | 1.44 | 0.68 | 2.30 | 1.22 | 2.39 | 1.66 ± 0.66 |
|  | 6 | 1.30 | 1.37 | 0.56 | 1.55 | 0.60 | 1.86 | 1.21 ± 0.52 |
|  | 8 | 1.66 | 0.90 | 1.06 | 0.95 | 0.73 | 1.87 | 1.19 ± 0.46 |
|  | 10 | 1.10 | 0.73 | 0.45 | 1.28 | 0.64 | 3.91 | 1.35 ± 1.29 |
|  | 24 | 0.38 | ND | 0.22 | 0.40 | 0.17 | 0.24 | 0.28 ± 0.10 |

ND: Failed to check out. *: Abnormal value, not calculated.

**Table S8.** Tissue concentrations of BAI at different time points after single TI and single IV of TRQ (0.12 mL/kg) in rats. (Mean ± SD, n=6)

| Admini-  stration | Time (h) | Concentrations (ng/kg) | | | | |
| --- | --- | --- | --- | --- | --- | --- |
|  |  | Lung | BALF | Trachea | Plasma | Brain |
| TI | 0.083 | 1302.25 ± 561.07 | 72.84 ± 44.98 | 52.9 ± 40.60 | 22.26 ± 7.63 | 7.93 ± 6.47 |
|  | 0.333 | 2796.49 ± 1722.19 | 226.18 ± 88.9 | 120.10 ± 114.83 | 53.30 ± 23.98 | 5.19 ± 2.04 |
|  | 1 | 2060.93 ± 913.51 | 126.16 ± 15.2 | 192.13 ± 125.04 | 43.07 ± 10.94 | 4.15 ± 1.48 |
|  | 4 | 52.25 ± 17.55 | 7.06 ± 2.83 | 26.62 ± 13.48 | 7.91 ± 3.30 | 3.17 ± 2.17 |
|  | 8 | 20.74 ± 4.24 | 1.87 ± 0.29 | 13.25 ± 5.17 | 7.74 ± 2.31 | 2.93 ± 0.14 |
| IV | 0.083 | 378.99 ± 277.71 | 1.28 ± 0.79 | 17.67 ± 6.23 | 1163.12 ± 877.25 | 7.35 ± 3.12 |
|  | 0.333 | 106.26 ± 69.02 | 1.08 ± 0.64 | 8.50 ± 0.58 | 575.16 ± 288.54 | 4.70 ± 1.06 |
|  | 1 | 25.47 ± 42.51 | 0.91 ± 1.43 | 1.49 ± 3.65 | 43.57 ± 18.99 | 3.78 ± 2.24 |
|  | 4 | 5.89 ± 3.26 | 0.12 ± 0.05 | ND | 22.45 ± 12.92 | 2.55 ± 0.17 |
|  | 8 | 4.55 ± 3.02 | 0.22 ± 0.08 | 2.83 ± 4.39 | 14.50 ± 11.77 | 3.04 ± 0.42 |

ND: Failed to check out.

**Table S9.** Tissues concentrations of ORO at different time points after single TI and single IV of TRQ (0.12 mL/kg) in rat. (Mean ± SD, n=6)

| Admini-  stration | Time (h) | Concentrations (ng/kg) | | | | |
| --- | --- | --- | --- | --- | --- | --- |
|  |  | Lung | BALF | Trachea | Plasma | Brain |
| TI | 0.083 | 70.89 ± 43.39 | 3.53 ± 2.28 | 19.63 ± 7.81 | 1.02 ± 0.50 | 0.64 ± 0.41 |
|  | 0.333 | 166.72 ± 113.10 | 10.76 ± 3.91 | 41.61 ± 26.27 | 2.19 ± 0.67 | 0.58 ± 0.16 |
|  | 1 | 102.41 ± 46.22 | 6.36 ± 1.55 | 40.38 ± 12.12 | 3.25 ± 0.73 | 0.57 ± 0.15 |
|  | 4 | 2.42 ± 0.61 | 0.03 ± 0.04 | 9.24 ± 2.06 | 1.18 ± 0.41 | 0.59 ± 0.73 |
|  | 8 | 1.23 ± 0.33 | ND | 2.30 ± 0.24 | 0.77 ± 0.43 | 0.70 ± 0.73 |
| IV | 0.083 | 27.28 ± 23.31 | 0.04 ± 0.03 | 8.15 ± 4.34 | 31.05 ± 24.49 | 0.91 ± 0.45 |
|  | 0.333 | 11.33 ± 7.53 | 0.01 ± 0.02 | 3.12 ± 0.89 | 15.76 ± 9.63 | 0.81 ± 0.25 |
|  | 1 | 1.76 ± 2.41 | ND | 1.69 ± 0.27 | 1.41 ± 2.13 | 1.96 ± 2.26 |
|  | 4 | 0.80 ± 0.23 | ND | 1.46 ± 0.12 | 1.15 ± 0.59 | 0.28 ± 0.02 |
|  | 8 | 0.69 ± 0.31 | ND | 1.63 ± 0.26 | 0.79 ± 0.43 | 0.49 ± 0.42 |

ND: Failed to check out.

**Table S10.** Pharmacokinetic parameters of BAI and ORO in the rat lung, BALF, trachea, brain and plasma after single TI and single IV of TRQ at the dose of 0.12 mL/kg (n=6).

| Analytes | Biological  matrix | Admini-  stration | PK parameters | | | | | | |
| --- | --- | --- | --- | --- | --- | --- | --- | --- | --- |
|  |  |  | T_1/2_  (h) | T_max_  (h) | C_max_  (ng/mL) | AUC_0-t_  (h·ng/mL) | AUC_0-∞_  (h·ng/mL ) | MRT_0-t_  (h) | MRT_0-∞_  (h) |
| BAI | Lung | TI | 1.02 | 0.33 | 2796.49 | 5502.07 | 5532.70 | 0.96 | 1.01 |
|  |  | IV | 1.46 | 0.08 | 378.99 | 188.23 | 197.81 | 1.19 | 1.62 |
|  | BALF | TI | 1.09 | 0.33 | 226.18 | 375.61 | 378.55 | 1.05 | 1.12 |
|  |  | IV | 2.75 | 0.08 | 1.28 | 3.26 | 4.14 | 2.20 | 4.29 |
|  | Trachea | TI | 1.87 | 1.00 | 192.13 | 535.83 | 571.58 | 1.78 | 2.34 |
|  |  | IV | 0.26 | 0.08 | 17.67 | 7.33 | 7.89 | 0.28 | 0.35 |
|  | Brain | TI | 6.99 | 0.08 | 7.93 | 27.86 | 57.38 | 3.52 | 11.02 |
|  |  | IV | 8.09 | 0.08 | 7.35 | 23.19 | 56.00 | 3.74 | 13.07 |
|  | Plasma | TI | 2.43 | 0.33 | 53.30 | 148.13 | 171.52 | 2.06 | 3.35 |
|  |  | IV | 1.33 | 0.08 | 1163.12 | 635.23 | 653.89 | 1.03 | 1.28 |
| ORO | Lung | TI | 1.03 | 0.33 | 166.72 | 286.94 | 288.77 | 0.93 | 0.99 |
|  |  | IV | 1.69 | 0.08 | 27.28 | 16.91 | 18.31 | 1.52 | 2.20 |
|  | BALF | TI | 0.47 | 0.33 | 10.76 | 17.27 | 17.31 | 0.79 | 0.80 |
|  |  | IV | —— | 0.08 | 0.06 | 0.02 | 0.02 | 0.17 | —— |
|  | Trachea | TI | 1.76 | 0.33 | 41.61 | 133.31 | 139.14 | 1.85 | 2.22 |
|  |  | IV | 4.86 | 0.08 | 8.15 | 13.96 | 24.33 | 3.42 | 8.36 |
|  | Brain | TI | 9.72 | 0.08 | 0.64 | 3.47 | 8.63 | 3.54 | 14.59 |
|  |  | IV | 4.16 | 0.08 | 0.91 | 3.70 | 5.38 | 2.75 | 6.25 |
|  | Plasma | TI | 3.46 | 1.00 | 3.25 | 12.79 | 16.65 | 2.75 | 5.12 |
|  |  | IV | 1.84 | 0.08 | 31.05 | 20.59 | 22.69 | 1.66 | 2.50 |

**
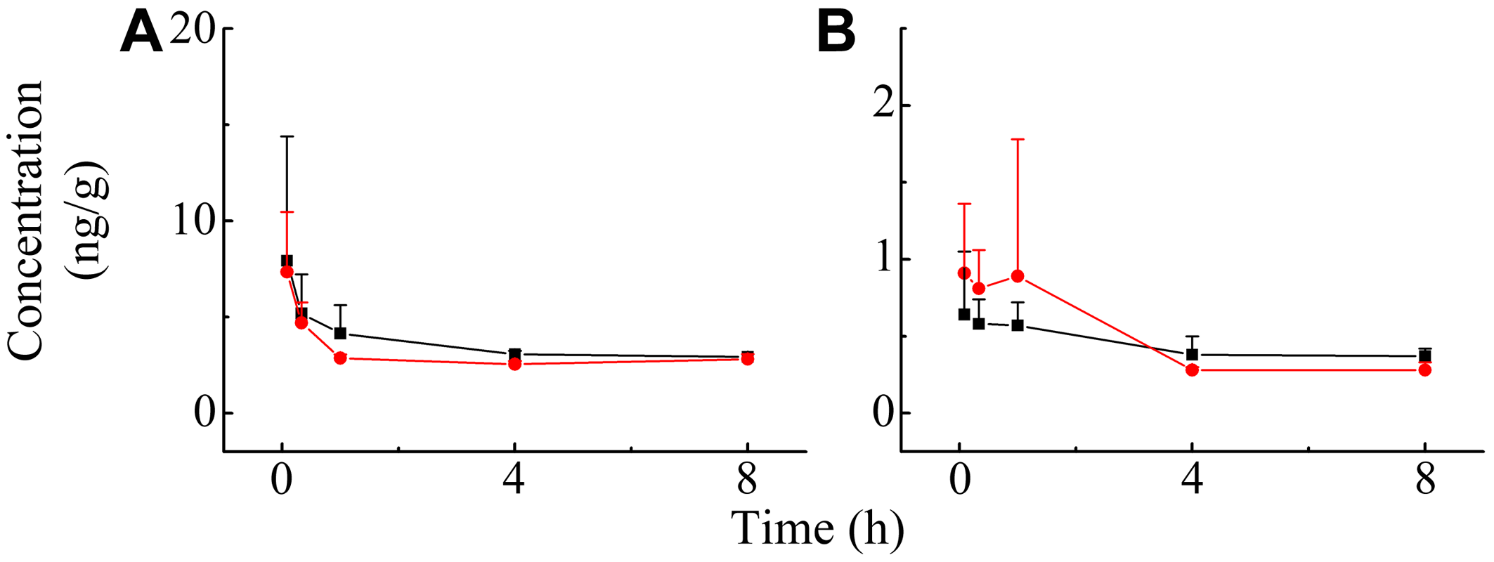
**

**Figure S1.** Mean concentration-time profiles of BAI (A) and ORO (B) in rat brain after TI (—■—) and IV (—●—) of TRQ at the dose of 0.12 mL/kg (n=6). Bars represent standard deviation.
